# Selective Inhibition of Bruton’s Tyrosine Kinase by a Designed Covalent Ligand Leads to Potent Therapeutic Efficacy in Blood Cancers Relative to Clinically Used Inhibitors

**DOI:** 10.1021/acsptsci.2c00163

**Published:** 2022-11-02

**Authors:** Bárbara B. Sousa, Cátia Rebelo de Almeida, Ana F. Barahona, Raquel Lopes, Ana Martins-Logrado, Marco Cavaco, Vera Neves, Luís A. R. Carvalho, Carlos Labão-Almeida, Ana R. Coelho, Marta Leal Bento, Ricardo M. R. M. Lopes, Bruno L. Oliveira, Miguel A. R. B. Castanho, Peter Neumeister, Alexander Deutsch, Gregory I. Vladimer, Nikolaus Krall, Cristina João, Francisco Corzana, João D. Seixas, Rita Fior, Gonçalo J. L. Bernardes

**Affiliations:** † Instituto de Medicina Molecular João Lobo Antunes, Faculdade de Medicina, Universidade de Lisboa, Avenida Prof. Egas Moniz, 1649-028, Lisbon, Portugal; ‡ Champalimaud Foundation, Avenida de Brasília, 1400-038, Lisbon, Portugal; § Yusuf Hamied Department of Chemistry, University of Cambridge, Lensfield Road, Cambridge CB2 1EW, U.K.; ∥ Centro Hospitalar Lisboa Norte, Department of Hematology and Bone Marrow Transplantation, Avenida Prof. Egas Moniz, 1649-035 Lisbon, Portugal; ⊥ Research Institute for Medicines (iMed.ULisboa), Faculdade de Farmácia, Universidade de Lisboa, 1600-277 Lisbon, Portugal; # Division of Hematology, Medical University of Graz, Auenbruggerplatz 38, 8036 Graz, Austria; ∇ Exscientia, The Schrödinger Building, Oxford Science Park, Oxford OX4 4GE, U.K.; ○ Centro de Investigación en Síntesis Química, Departamento de Química, Universidad de La Rioja, 26006 Logroño, Spain; ◆ TARGTEX S.A., Avenida Tenente Valadim, N°17, 2F, 2560-275 Torres Vedras, Portugal

**Keywords:** covalent inhibitor, BTK, antitumor activity, preclinical studies, hematological cancers

## Abstract

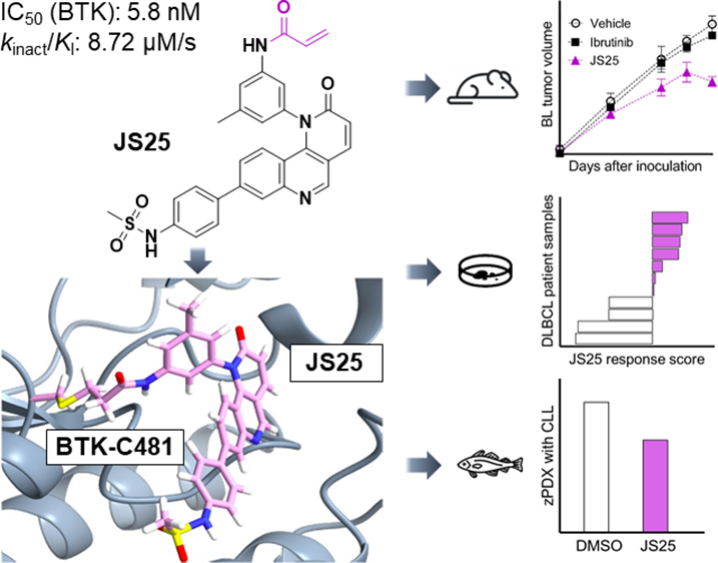

Bruton’s tyrosine kinase (BTK) is a member of the TEC-family kinases and crucial for the proliferation and differentiation of B-cells. We evaluated the therapeutic potential of a covalent inhibitor (JS25) with nanomolar potency against BTK and with a more desirable selectivity and inhibitory profile compared to the FDA-approved BTK inhibitors ibrutinib and acalabrutinib. Structural prediction of the BTK/JS25 complex revealed sequestration of Tyr551 that leads to BTK’s inactivation. JS25 also inhibited the proliferation of myeloid and lymphoid B-cell cancer cell lines. Its therapeutic potential was further tested against ibrutinib in preclinical models of B-cell cancers. JS25 treatment induced a more pronounced cell death in a murine xenograft model of Burkitt’s lymphoma, causing a 30–40% reduction of the subcutaneous tumor and an overall reduction in the percentage of metastasis and secondary tumor formation. In a patient model of diffuse large B-cell lymphoma, the drug response of JS25 was higher than that of ibrutinib, leading to a 64% “on-target” efficacy. Finally, in zebrafish patient-derived xenografts of chronic lymphocytic leukemia, JS25 was faster and more effective in decreasing tumor burden, producing superior therapeutic effects compared to ibrutinib. We expect JS25 to become therapeutically relevant as a BTK inhibitor and to find applications in the treatment of hematological cancers and other pathologies with unmet clinical treatment.

Bruton’s tyrosine kinase (BTK) belongs to the TEC family of cytoplasmatic kinases and presents a functional cysteine in the 481 position prone to covalent binding.^1^ BTK is expressed in many cells of the hematopoietic lineage, including B- and T-cells, monocytes, neutrophils, and mast cells.^1,2^ Expression of this protein is essential for the development and function of mature B-cells, and inactivating mutations in the BTK gene cause primary immunodeficiency disease X-linked agammaglobulinemia in humans and X-linked immunodeficiency in mice.^3^ Moreover, constitutive activation of BTK in systemic lupus erythematosus results in an accumulation of antibody-secreting plasma cells.^4^ BTK is also a proximal component of the B-cell receptor, and it is activated by upstream Src-family kinases through intermediate signaling generated by PI3 kinase.^5,6^ Once activated, BTK phosphorylates phospholipase-Cγ (PLCγ), leading to Ca^2+^ mobilization and activation of NF-κB and MAP kinase pathways, promoting proliferation and survival of B-cells.^7^ BTK also induces the dependent proinflammatory production of cytokines IL-6 and IL-10^8,9^ and controls integrin-mediated adhesion of B-cells^10^ and their responses to chemokines, such as SDF-1.^11,12^ Deregulation of BTK is observed in some autoimmune diseases^13,14^ and in hematological cancers, including myeloid and B-lymphocytic leukemias (acute myeloid leukemia (AML), acute lymphocytic leukemia (ALL), chronic lymphocytic leukemia (CLL)), Waldenström’s macroglobulinemia (WM), mantle cell lymphoma (MCL), Burkitt’s lymphoma (BL), and diffuse large B-cell lymphoma (DLBCL), further indicating that BTK is an effective target for numerous pathologies.^1,15,16^

Since its first description, multiple BTK inhibitors (BTKi) have been developed. The irreversible BTK inhibitor ibrutinib was the first FDA-approved BTKi and is associated with high response rates in relapsed/refractory CLL, WM, and MCL and in chronic graft *versus* host disease.^17,18^ However, with a broad selectivity profile, ibrutinib inhibits the whole TEC family, EGFR, JAK3, Her2, Blk, and ITK kinases. Ibrutinib’s “off-target” binding is usually associated with adverse effects such as rash, diarrhea, bleedings, infections, and atrial fibrillation, leading to treatment withdrawal in 9–23% of patients.^19^ Ibrutinib can also antagonize rituximab-induced antibody-dependent cellular cytotoxicity due to inhibition of its family member ITK, further limiting its use in combination regimens.^20^ Despite the clinical success of ibrutinib, further refinement was required in terms of adverse effects, fueling the development of highly selective BTKi. Acalabrutinib and zanubrutinib are the most recently FDA-approved inhibitors and show clinical potential with improved selectivity and with fewer adverse effects relative to ibrutinib. Acalabrutinib was approved in 2017 for MCL and in 2019 for CLL. With higher selectivity than ibrutinib, acalabrutinib inhibits only BTK, TEC, BMX, and TXK.^19,21^ Zanubrutinib was approved in 2019 to treat MCL in adults who previously received therapy.^19,22^ Zanubrutinib is similar to acalabrutinib with less activity on TEC and ITK and also displays higher potency and selectivity for BTK than ibrutinib, with fewer “off-target” effects. In this study, we investigate the therapeutic potential of a small covalent molecule (JS25) with nanomolar potency against BTK (5.8 nM). JS25 was obtained from the scaffold of BMX-IN-1, a recently discovered molecule that has been shown to also inhibit BTK, as part of our efforts to identify regions of the molecule that could be modulated for improved efficacy and selectivity.^23,24^ Initially, we had explored the JS25 potential for treating prostate cancer, but later experiments revealed that JS25 was highly selective for BTK, and therefore, it could have therapeutic importance in blood malignancies that derive from BTK’s abnormal expression. Following the preliminary data, we sought to characterize the binding mode of JS25 to BTK and asserted its selectivity against a panel of eight kinases related to BTK’s signaling pathway or with an equally placed cysteine as to the Cys481 of BTK. We further demonstrate that the lead compound has potential to inhibit the proliferation of several hematological cancers and to induce the degradation of BTK. Validation of its therapeutic effect was conducted in xenograft murine models of Burkitt’s lymphoma, and in patient-derived models of diffuse large B-cell lymphoma and chronic lymphocytic leukemia. Finally, we explore the capability of JS25 to cross the brain–blood barrier and treat infiltration of tumor cells in the brain.

## Experimental Section

### Putative 3D Structure of JS25 Linked to BTK

#### Docking Studies with AutoDock 4.2

AutoDock 4.2^25^ was used to predict the region where JS25 binds to BTK (PDB: 6TFP). Standard settings for *autogrid* (number of grid points in *xyz*: 126, 126, 126; spacing (Å) = 0.375) and *autodock* (genetic algorithm, max. number of evaluations = 250,000, output = Lamarckian GA(4.2)) were selected with AutoDockTools 1.5.6.

#### Molecular Dynamics (MD) Simulations

Simulations on JS25 or ibrutinib bound to BTK were performed with the AMBER 20 package (University of California) and implemented with the GAFF2 force field.^26^ For the BTK/ibrutinib complex, the coordinates of the reported X-ray structure were used as starting coordinates (PDB: 5P9J). The setup for the molecular dynamics was performed as previously described,^27^ with the production step set to 500 ns.

### Selectivity Determination against BTK

In-cell target engagement was performed at Reaction Biology Corporation, using NanoBRET technology. Very briefly, HEK296T cells were transfected and treated in duplicate with JS25 for 1 h of incubation. The compound was diluted 10 times with 3-fold dilution, starting at 1 μM. Curve fits were performed only when the % NanoBret signal at the highest concentration of compounds was less than 55%. The IC_50_ values were determined using GraphPad Prism 8 (GSL Biotech LLC).

### Inhibition Kinetics Characterization

The BTK enzyme system and the ADP-Glo kinase assay were purchased from Promega Corporation (V2941). Ibrutinib was acquired from BOC Sciences, and acalabrutinib from Advanced ChemBlock. In each kinase reaction, the concentration of BTK was set to 4 ng/μL. The peptide substrate Poly (4:1 Glu, Tyr) and ATP concentrations were set to 0.25 mg/mL and 50 μM, respectively. BTK was preincubated with different inhibitor concentrations (8-fold serial dilutions, starting at 100 nM) over different time periods (2–60 min), before initiating the kinase reactions. Reactions were started by adding a 2.5× Poly E4Y1/ATP mixture. The reactions were carried out in a 384-well plate and quenched simultaneously with the addition of 5 μL of the ADP-Glo reagent to consume the remaining ATP within 40 min. Then, 10 μL of the kinase detection reagent was added into the wells and incubated for 30 min to produce a luminescence signal. The signal was measured using an Infinite M200 Microplate Reader (Tecan) with an integration time of 0.250 s. The observed rate constants for inhibition (*k*_obs_) at different inhibitor concentrations were determined from the slope of a semilogarithmic plot of inhibition *versus* time and replotted against inhibitor concentration (nM). The experimental values were fitted into a hyperbolic function using GraphPad Prism 8 to obtain *K*_I_, *k*_inact_, and *k*_inact_/*K*_I_, as described previously.^28^

### Cell Culture

All cell lines were purchased from ATCC, except for DoHH-2 cells that were obtained from DSMZ. Cells were cultivated in complete DMEM supplemented with 10% (vol/vol) FBS (Gibco) and 1% of penicillin/streptomycin (Gibco). The cells were grown in a humidified atmosphere of 5% CO_2_ at 37°C, with the medium changed every other day.

### Cell Viability Assay

Cells were inoculated at a density of 5000 cells/well. Serially diluted compounds (starting at 50 μM) were added 24 h later. The assay was performed in triplicates. After 72 h of incubation, cellular viability was assessed by CellTiter-Glo (Promega) according to the manufacturer’s instructions. The values were normalized with the vehicle (DMSO), and the IC_50_ was calculated using GraphPad Prism 8.

### *In Vitro* Analysis of the Blood–Brain Barrier Permeability

Human cerebral microvascular endothelial cells (HBEC-5i) were cultured as a monolayer on attachment factor protein solution (AF)-coated T-flasks (Gibco), using DMEM/F12 medium (Gibco), supplemented with 10% FBS, 1% penicillin/streptomycin, and 40.0 μg/mL endothelial cell growth supplement (ECGS, Sigma), according to the manufacturer’s instructions. The capacity of the compounds to cross the brain–blood barrier (BBB) was evaluated using an *in vitro* HBEC-5i cell model, as previously described.^29^ Samples from the apical and basolateral sides were collected, and fluorescence intensity was measured using a Varioskan LUX multimode microplate reader. The retention was considered the difference between the initial fluorescence of compounds (100%) and the aggregated apical and basolateral fluorescence.

### BTK Degradation in Raji Cells (Burkitt’s Lymphoma)

Raji cells were inoculated at 0.5 × 10^6^ cells/mL, and JS25 was added at a final concentration of 10 μM. At 0, 4, and 15 h of incubation, the cells were harvested, and the pellets were resuspended in lysis buffer (20 mM Tris–HCl, 150 mM NaCl, pH 8.0, 0.1% Triton X-100), supplemented with EDTA-free Protease Inhibitor Cocktail (Merck) and DNase I (Merck). The protein concentration was determined using the Pierce BCA Protein Assay Kit (Thermo Scientific). Western blot was performed with rabbit BTK antibody (1:1000; 3533, Cell Signaling Technology), mouse α-Tubulin antibody (1:5000; 5168, Merck), goat anti-rabbit IgG (H + L) secondary antibody HRP (1:7000; 65-6120, Invitrogen), and goat anti-mouse IgG (H + L) secondary antibody HRP (1:5000; 2-6520, Invitrogen). The Signal was revealed with the Clarity Western ECL Substrate (Bio-Rad Laboratories), and band intensity was measured using ImageJ software (National Institutes of Health).

### Mice Xenograft Model of Burkitt’s Lymphoma

Female adult BALB/c/NSG mice were injected subcutaneously with 1 × 10^6^ Raji cells, in a 1:1 solution of Matrigel Matrix (Corning) to create solid tumors. When tumors reached 180 mm^3^ on average, mice were randomized into four groups (*n* = 6/group), and dosing began every 2 days. JS25 and ibrutinib were administered *via* i.p. injection, as a mixture of 20% of Kolliphor (Sigma-Aldrich) in PBS. Three treatment groups were included based on a similar study reported by Li et al.: one dose of ibrutinib (10 mg/kg), and two doses of JS25 (10 and 20 mg/kg). Tumor size and body weight were monitored periodically for 12 days. At the end of the experiment, mice necropsies were performed. Stereological analysis was conducted by the Histopathology Unit at Instituto Gulbenkian de Ciência and by the Comparative Pathology Unit at Instituto de Medicina Molecular João Lobo Antunes. Quantification of metastases and cell necrosis was performed in all groups (*n* = 5/group). Statistical analysis was conducted using one-way ANOVA. The Dunnet test was used to analyze the statistical significance between the treatment groups and the control.

### *Ex Vivo* Model of Diffuse Large B-Cell Lymphoma

#### Primary Material Collection and Purification

Primary lymph node samples were taken from patients, following hospital standard operating procedures. Clinical information including diagnosis was collected by the study center in a case report form. Target markers were confirmed by flow cytometry at the final laboratory prior to use.

#### Cell Plating, Assay, and Screening

Cells were plated at 10,000–20,000 cells per well in 384-well PerkinElmer Cell Carrier Ultra plates, containing prespotted small molecules in DMSO distributed by a Labcyte ECHO, in quadruplicate technical replicates in 4-point dose–response curves starting at 10 μM and decreasing by 1:3. DMSO volume in each well including controls were kept constant at 0.1% final volume of media. Plates were randomized and contained at least 15 DMSO vehicle control wells. Incubation took place for 72 h at 37 °C in air supplemented with 5% CO_2_. At the end of the incubation period, the cells were stained with a viability dye (Invitrogen), fixed, and permeabilized using low-concentration formaldehyde and Triton X-114 in DPBS, and the resulting monolayers were stained with fluorescent antibodies against surface markers (CD19 (eBiosciences, clone HIB19), CD20 (BD, clone L27), and CD79a (BioLegend, clone HM47) along with DAPI (Sigma)). Fluorescent antibodies are used in different nonoverlapping fluorescent channels.

#### Imaging and Image Analysis

Imaging of the primary cell monolayer was performed using PerkinElmer CLS spinning disk automated confocal microscopes, with nonoverlapping, sequential, fluorescent channel imaging. All images were taken with a 20× objective. Five fields were imaged, representing at least 50% of the well bottom, for each well (seven TIFF images in total per field, one for each color channel plus brightfield, and 405 nm for DAPI, and merged). For analysis, the images were subject to image illumination correction. Cell identification in each image works by finding the cell nucleus (relying on DAPI staining) using classical thresholding approaches. Segmentation was performed using proprietary algorithms. Classification (cell antigen expression and viability) of every single cell was achieved using deep convolutional neural networks trained on B-cells and other cells from B-NHL samples stained with the specific markers utilized for these experiments, as well as on the fixable live/dead viability dye. The network considers variations in staining of the marker and viability marker intensity (cytoplasm and membrane localized) along with other stain-based characteristics. For this work, the networks had at least 95% classification accuracy. Information on the calculation of the drug response score (DRS), the relative cell fraction, and cell fraction can be found in Snijder et al. All raw Pharmacoscopy data were visualized in R (3.6.1).

### Zebrafish Xenograft Model of Chronic Lymphocytic Leukemia

#### Peripheral Blood Mononuclear Cell (PBMC) Isolation and Cryopreservation

Whole blood (3–6 mL) from CLL patients (Supporting Table S1) was collected, and PBMCs were purified by Ficoll-Paque PLUS (GE Healthcare) density centrifugation.

#### PBMC Processing for Zebrafish Injection

The collected PBMCs were resuspended in RPMI (Biowest) with 3 times their volume and centrifuged at 1400 rpm, 4 °C, for 7 min. Cell pellets were resuspended in DPBS 1× (Biowest) supplemented with universal nuclease at 25 U/mL (Thermo Scientific). Concentration was normalized to 5 × 10^6^ cells/μL for zebrafish patient-derived xenograft (zPDX) generation. Prior to drug efficacy analysis in zebrafish, the cells were distributed to proceed for flow cytometry, to determine the percentage of CD19+CD5+ cells within the CD45+ population, from PBMCs of each CLL patient. The maximum tolerated concentration was also determined for each compound in noninjected zebrafish larvae (Supporting Figure S1).

#### Zebrafish Patient-Derived Xenograft Injection and Drug Administration

Zebrafish larvae were anesthetized with Tricaine 1×, and thawed PBMCs were microinjected into the perivitelline space of anesthetized zebrafish larvae at 48 h post fertilization. After injection, zPDXs were sorted and randomly distributed into the different treatment groups in E2 medium/DMSO (control), JS25, ibrutinib, and venetoclax. zPDXs were maintained at 34 °C, and all drugs were renewed daily for 2 consecutive days. At the end of the assay, 4 days post injection, zebrafish xenografts were sacrificed with an overdose of Tricaine 25× and fixed in 4% formaldehyde (Thermo Scientific) overnight, followed by storage in 100% methanol (VWR) at −20 °C.

#### Whole Mount Immunofluorescence

The whole mount immunofluorescence protocol was started by rehydrating the xenografts through methanol series (75% > 50% > 25% in PBS 1×-Triton 0.1%). Next, the xenografts were permeabilized in PBS 1× with 0.1% (v/v) Triton and incubated in a blocking solution (containing 1% BSA and 1.5% goat serum) for 1 h at room temperature. The xenografts were incubated with the primary antibodies (anti-cleaved caspase 3—Cell Signaling Technology, clone Asp175, 9661, 1:100; anti-human mitochondria—Merck Millipore, clone 113-1, MAB1273, 1:50) diluted in the blocking solution overnight at 4 °C, followed by additional overnight incubation with 1:400 of secondary antibodies: Alexa goat anti-rabbit 594 (35560, Thermo Scientific) and Alexa goat anti-mouse 647 (84545, Thermo Scientific), and nuclei counterstaining with DAPI at 50 μg/mL (Sigma-Aldrich).

#### Imaging and Quantification

All images were obtained using a Zeiss LSM 980 Upright confocal laser scanning microscope. Xenografts were mounted in in-house Mowiol mounting media, and sequential images along tumor’s depth (from cloaca until the end of the tail) with a 5 μm interval were acquired using the z-stack function. Upon image acquisition, analysis was performed using ImageJ software. For tumor burden, the area occupied by the PBMCs in each slice of the z-stack pile was determined by ImageJ software and summed up to obtain the tumor burden per xenograft. To express the outcome as fold induction, values obtained for controls and treatment conditions were normalized to the control. Tumor incidence was given by dividing the number of zebrafish xenografts that presented tumor cells between cloaca and the end of the tail, per the total number of zebrafish xenografts alive at the end of the assay (2 days post injection).

#### Statistical Analysis of Zebrafish Patient-Derived Xenograft Data

Statistical analysis was performed using GraphPad Prism 8. All data were challenged by two normality tests—the D’Agostino–Pearson and Shapiro–Wilk normality tests. A Gaussian distribution was only assumed for data sets that pass both normality tests and were analyzed by an unpaired *t*-test with Welch’s correction. By opposition, data sets that did not pass one or both normality tests were analyzed by the Mann–Whitney test, an unpaired and nonparametric U test. Fisher’s exact test was used for tumor incidence analysis.

## Results

### JS25 Exhibits Higher Potency in Inhibiting BTK Compared to Ibrutinib, Acalabrutinib, and BMX-IN-1

**Figure 1:**
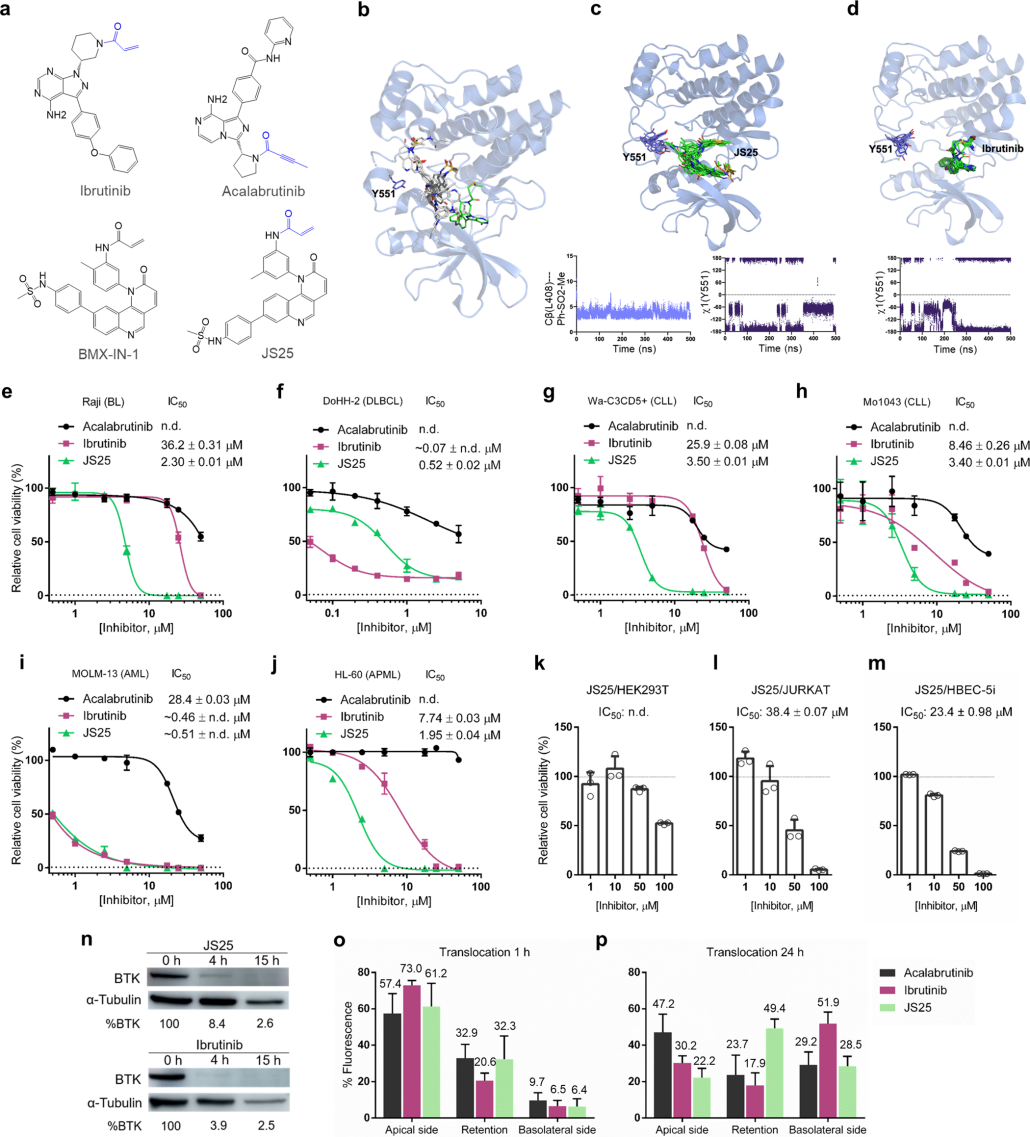
Putative structure of BTK covalently inhibited and cell viability assays. (a) Chemical structure of JS25 and other BTK inhibitors used. (b) The energetically best poses for BTK as determined by docking calculations. (c) Overlay of 10 frames of BTK/JS25 complex sampled from 0.5 μs MD simulations, together with the distance between the sidechain of Leu408 and the aromatic ring (Ph-SO_2_Me) of JS25, and the geometry of the sidechain (χ^1^ dihedral angle) of Tyr551 throughout MD simulations. (d) Overlay of 10 frames of BTK/Ibrutinib complex sampled from 0.5 μs MD simulations, together with the geometry of the sidechain (χ1 dihedral angle) of Tyr551 through MD simulations. BTK is shown as blue ribbons, and carbon atoms of the ligand and Tyr551 are shown in green and purple, respectively. (e) Cell viability of Raji, (f) DoHH-2, (g) WA-C3CD5+, (h) Mo1034, (i) MOLM-13, (j) HL-60, (k) HEK293T, (l) JURKAT, and (m) HBEC-5i. Cells were treated with serial doses of acalabrutinib, ibrutinib, and JS25 for 72 h. Error bars correspond to the standard deviation of the mean, *n* = 3 technical replicates. (n) Degradation analysis of BTK after treating Raji cells with JS25. (o) Translocation profile of different compounds (15 μM) at 1 h and (p) 24 h. Experiments were performed in triplicates on at least three different days using independently grown cell cultures. Error bars correspond to the standard deviation of the mean.

Covalent modification of BTK is a two-step process that covers the affinity of the initial noncovalent binding, *K*_I_, and the rate of covalent bond formation, *k*_inact_.^30^ The rate of inactivation (*k*_inact_/*K*_I_) is a second-order event, which describes the efficacy of the covalent bond binding event. To characterize the covalent interactions of JS25 with BTK, evaluation of the irreversible binding efficacy was performed as previously described.^23^ Additionally, we included ibrutinib, acalabrutinib, and BMX-IN-1 (Figure 1a). The calculated kinetic parameters *K*_I_*, k*_inact_, and *k*_inact_/*K*_I_ are shown in Table 1. The data demonstrated similar binding affinity between JS25, ibrutinib, and BMX-IN-1 for BTK, as indicated by their respective *K*_I_ values: 0.77, 0.59, and 1.29 nM. Out of the four compounds, acalabrutinib presented the weakest binding affinity for BTK (*K*_I_ = 15.07 nM). Most importantly, the rate of covalent bond formation, *k*_inact_, of JS25 is 10-fold faster (0.401 min^–1^) compared with ibrutinib (0.041 min^–1^), acalabrutinib (0.038 min^–1^), and BMX-IN-1 (0.038 min^–1^); consequently, JS25 efficiently inactivated BTK with a *k*_inact_/*K*_I_ of 8.72 μM^–1^ s^–1^, displaying an increased rate of inactivation of approximately 8-fold relative to ibrutinib (1.17 μM^–1^ s^–1^), 200-fold relative to acalabrutinib (0.04 μM^–1^ s^–1^), and 18-fold relative to BMX-IN-1 (0.49 μM^–1^ s^–1^). The differences in kinetic properties between the tested compounds highlight the variances in their specific binding modes and suggest an improved complementarity of JS25 with the target protein.

**Table 1:** Comparison of the Kinetic Parameters

compound	*K*_I_ [nM]	*k*_inact_ [min^–1^]	*k*_inact_/*K*_I_ [μM^–1^ s^–1^]
JS25	0.77 ± 0.06	0.401 ± 0.064	8.72 ± 1.02
ibrutinib	0.59 ± 0.03	0.041 ± 0.004	1.17 ± 0.13^a^
acalabrutinib	15.07 ± 0.51	0.038 ± 0.005	0.04 ± 0.01^b^
BMX-IN-1	1.29 ± 0.50	0.038 ± 0.008	0.49 ± 0.15^c^

a Value with a 0.17 deviation from published results (Liclican et al., 2020).^48^

b Value with a 0.002 deviation from published results (Liclican et al., 2020).^48^

c Value with a 0.29 deviation from published results (Wang et al. 2017).^49^

### Selectivity and Inhibition for BTK are Induced by Hijacking of Me477, Leu408, and Tyr551

The putative 3D structure of JS25 covalently bound to BTK was generated. AutoDock 4.2 software was used to predict the region where JS25 binds to BTK (noncovalent docking). The crystal structure of this protein, reported together with an inhibitor (PDB: 6TFP), was used for the docking studies. Interestingly, the best 10 docking poses in terms of binding affinity interact with BTK in the same region as other reported inhibitors^21^ (Figure 1b). A detailed analysis of the different poses shows that pose #10 localizes the Michael acceptor moiety near Cys481. Therefore, we covalently bound JS25 with this 3D orientation to this cysteine residue of BTK and performed 0.5 μs MD simulations in explicit water (Figure 1c). The simulations show that the complex is stable due to the occurrence of hydrogen bonds and hydrophobic contacts between the ligand and the receptor. Hydrogen bonds are established between the oxygen atoms of the sulfonamide and the main chain of Me477 (which occupies about 30% of the total trajectory time). Equally, the aromatic ring containing the sulfonamide group is engaged in a CH/π interaction with the sidechain of Leu408, which is maintained throughout the simulation time (Figure 1c). We also analyzed the dynamics of Tyr551, as BTK inhibitors can be classified according to their ability to trigger the “sequestration” of this Tyr residue. In cells, sequestration of Tyr551 was shown to render it inaccessible for phosphorylation.^21^ According to our calculations, Tyr551 is sequestrated around 60% of the whole trajectory (χ^1^ torsional angle close to 180°). To validate our simulation protocol, we performed MD simulations for the complex of BTK with ibrutinib (Figure 1d), using the X-ray structure as the initial coordinates (PDB: 5P9J). As in the X-ray structure, the MD simulations show a hydrogen bond between the −NH_2_ group of the ligand and the main chain of Glu475 (with a population of about 32%) and a hydrophobic contact between the phenyl group of ibrutinib and (population about 83%). As for the dynamics of Tyr551, our calculations showed that this residue is inaccessible about 72% of the time, which is consistent with the X-ray structure and experimental data.

### JS25 Presents a More Favorable Selectivity Profile than Ibrutinib and Acalabrutinib

Compound selectivity is a crucial factor to take into consideration in drug discovery, as in many cases, a lack of selectivity can translate into increased toxicity in clinical trials.^31^ It is also important to note that selectivity toward specific TEC kinases and other pathway-related proteins is particularly difficult, as these share a high sequence and structural similarity, including a reactive cysteine in the catalytic pocket.^32^ To determine whether JS25 is a selective binder, we evaluated its inhibitory capability against BTK, BMX, ITK, TXK, and TEC and against other BTK pathway-related proteins (BLK, EGFR, ERBB2, and JAK3). The selectivity of JS25 is shown in Table 2, and it is defined as IC_50_ kinase/IC_50_ BTK. JS25 showed an IC_50_ value of 28.5 nM against BTK, and the value for BMX was 49.0 nM, representing an ∼2-fold increase in the selectivity toward BTK. Within the TEC-family kinases, JS25 presented ∼7-fold, ∼8-fold, 15-fold, and 100-fold higher selectivity for BTK, relative to TXK, TEC, ITK, and BLK, respectively. Importantly, the values of IC_50_ for EGFR, ERBB2, and JAK3 were all higher than 3 μM. Overall, our data reveal that JS25 is very selective for both BMX and BTK, but with lower reactivity for other proteins within the TEC family, as well as for other proteins in BTK’s signaling pathways, possibly mitigating the chances for “off-target” effects in the clinical stages.

**Table 2:** Kinome Selectivity of JS25

kinase	IC_50_ (M)^a^^b^	selectivity (kinase/BTK)
BTK	2.85 × 10^–8^ ± 0.55	1
BMX	4.90 × 10^–8^ ± 0.40	1.7
TXK	1.90 × 10^–7^ ± 0.50	6.7
TEC	2.20 × 10^–7^ ± 0.30	7.7
ITK	4.40 × 10^–7^ ± 0.10	15.4
BLK	2.60 × 10^–6^ ± n.d.	104
EGFR	>3 × 10^–6^	n.d.
ERBB2	>3 × 10^–6^	n.d.
JAK3	>3 × 10^–6^	n.d.

a Average of duplicates, showing mean ± S.D.

b n.d.: not determined.

### JS25 Has a Wide Spectrum of Activity in Blood Cancer Cell Lines

Having demonstrated JS25 as a potent inhibitor of BTK in biochemical assays, we turned to its characterization in standard cell lines of hematological cancers, related to an abnormal expression of BTK, including BL, DLBCL, CLL, AML, and acute promyelocytic leukemia (APML). Acalabrutinib and ibrutinib were also included to validate the therapeutic relevance of JS25 in these cell lines. The results presented in Figure 1e–m show that JS25 has a significant effect on viable cell growth in all of the tested cells, and it has the capability to inhibit the proliferation with similar or greater potency than the FDA-approved BTKi, acalabrutinib, and ibrutinib. JS25 presented 15-fold greater efficacy than Ibrutinib to inhibit the proliferation of Raji cells (BL), with an IC_50_ value of 2.3 μM (Figure 1e). In DoHH-2 (DLBCL), Mo1043 (CLL), and MOLM-13 (AML) cell lines, there were no major improvements (Figure 1f,h,i); however, in WA-C3CD5+ cells (CLL), the antiproliferative potency of JS25 (3.5 μM) was approximately 7-fold greater than Ibrutinib (25.9 μM; Figure 1g). In addition, JS25 also presented better efficacy than ibrutinib in HL-60 cells (APML) with an IC_50_ value of 1.95 μM (Figure 1j). Importantly, other non-B-cell lines (JURKAT, HEK293T, and HBEC-5i) were not as sensitive to the treatment (Figure 1k–m). Degradation of BTK was also investigated by treating wild-type Raji cells with a 10 μM concentration of JS25 and ibrutinib. Western blot analysis showed that BTK degradation was evident at 4 h of treatment, and it was almost completed at 15 h (Figure 1n). These results validate JS25 as a potential therapeutic candidate with applicability against hematological cancers and demonstrate its ability to inhibit both the catalytic activity and the expression of BTK in tumor cells.

### JS25 Effectively Crosses the Blood–Brain Barrier

In several types of blood cancers, infiltration of malignant white blood cells occurs in the central nervous system (CNSi) and is only detected in 3–5% of patients at initial diagnosis, and 30–40% of patients at relapse.^33^ In relapsed and refractory MCL CNSi, monotherapy with BTKi has been proven to be effective, with an objective response rate of 68%. This efficacy is attributed to the ability of these drugs to cross the BBB and reach the tumor site.^34^ The permeability of JS25 on the BBB was evaluated using an *in vitro* HBEC-5i cell model. Ibrutinib and acalabrutinib were included as controls. As shown in Figure 1o,p, JS25 and acalabrutinib showed similar permeability to the BBB at 24 h (28.5 and 29.2%, respectively). However, these were comparatively lower than the permeability of ibrutinib (51.9%), due to higher retention rates of JS25 and acalabrutinib in the cells. Depending on the intracellular mechanism involved, higher retention of the compound in the BBB could result in its degradation or in greater durability of the treatment. These results open a possibility for JS25 to become useful for the treatment of more aggressive forms of hematological cancers.

### JS25 Has a Superior Therapeutic Effect Relative to Ibrutinib in a Xenograft Model of BL

**Figure 2:**
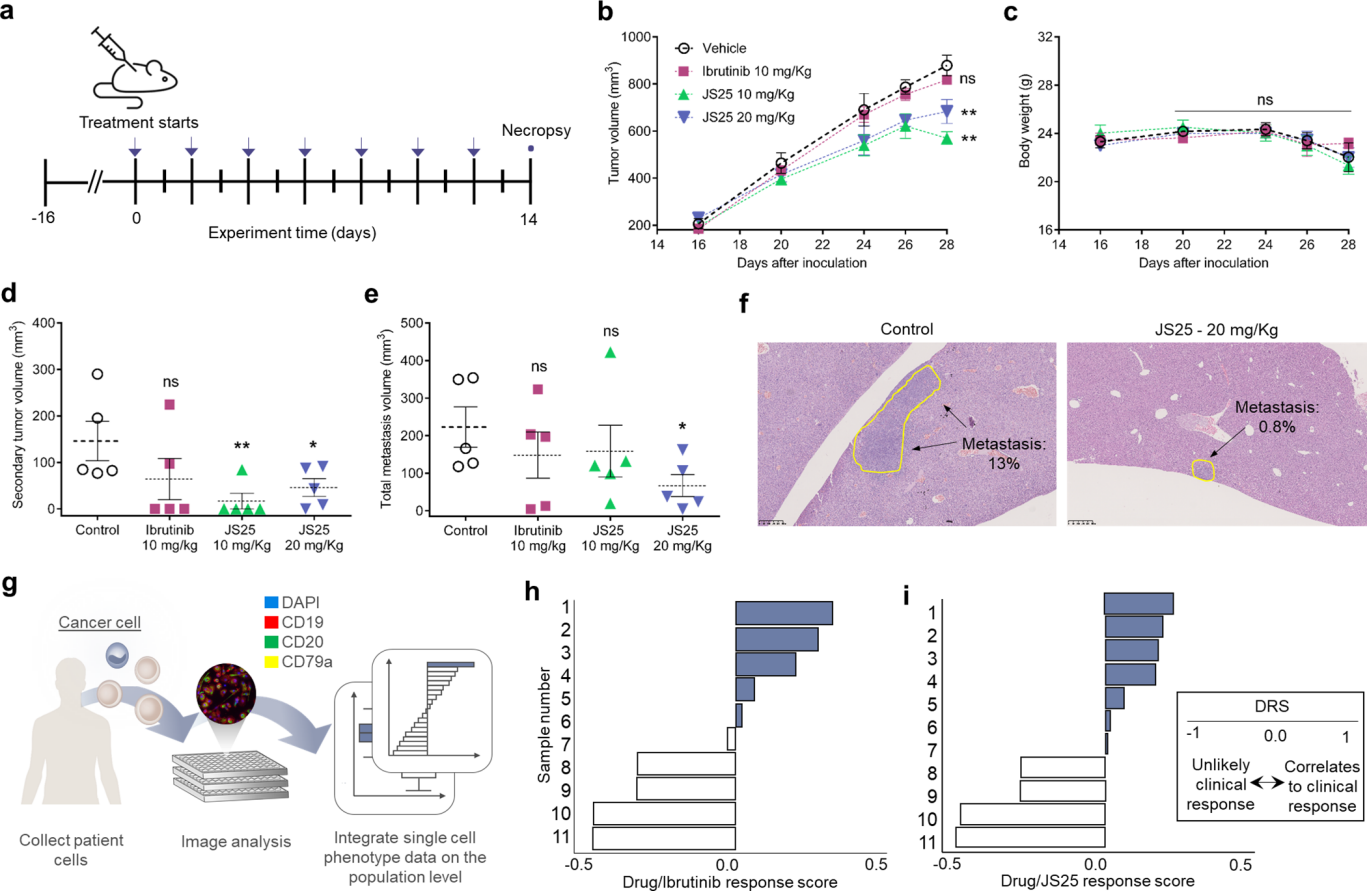
JS25 treatment inhibits the tumor growth of Burkitt’s lymphoma and induces selective *ex vivo* cytotoxicity in primary DLBCL samples. (a) Schematic representation of the *in vivo* assay. Blue arrows indicate days of treatment. (b) Tumor size and (c) body weight were monitored periodically. (d, e) Quantification and analysis of the metastases and tumor formation observed (*n* = 5/group), ((b) **p = 0.0018 and 0.0090, (d) **p = 0.0086, *p = 0.0418, (e) *p = 0.0386). Statistical analysis was conducted by one-way ANOVA, followed by Dunnett’s test for significance: not significant (ns) *p* > 0.05; *p < 0.05 (*); **p < 0.01. (f) Example of neoplastic cells observed in the liver of the control and JS25-treated groups. (g) Schematic representation of the *ex vivo* experiment. (h) JS25 and (i) ibrutinib/drug response score (DRS) calculated as 1-mean of the RCF. Each concentration point for each sample was performed in four replicates at 72 h incubation time point. Blue indicates DRS > 0, and white indicates DRS < 0. DRS scores > 0 indicate “on-target” cytotoxic response, and <0 indicates general cytotoxicity or “off-target” cytotoxic response.

To further validate its therapeutic potency, JS25 was examined in a mouse xenograft model inoculated subcutaneously with human lymphoma Raji cells (BL). This study comprised a vehicle control group and three treatment groups, including one dose of ibrutinib (10 mg/kg) and two doses of JS25 (10 and 20 mg/kg). The compounds were administered through intraperitoneal injection once every two days for 14 days, and tumor sizes were measured periodically (Figure 2a). As shown in Figure 2b, JS25 caused a significant reduction in the solid tumor sizes (around 30–40%), while ibrutinib–treated groups did not show significant changes relative to the control. Additionally, no weight fluctuations were observed by the end of the treatment (Figure 2c). Considering that drug dosing strongly influences the existing number of metastases, we sought to determine the overall percentage in each experimental group (Figure 2d–f and Supporting Table S2). Our quantitative analysis revealed that mice treated with JS25 had a significant reduction in their secondary tumor formation (71–88%; Figure 2d); however, only mice treated with the highest dose of JS25 (20 mg/kg) presented a significantly lower percentage of metastases (70% reduction; Figure 2e–f and Supporting Table S2). For both ibrutinib and JS25 (10 mg/kg doses), the reduction was similar and around 30% (Figure 2e). Infiltration of tumor cells was not observed in the heart and kidneys, and drug-induced necrosis of normal cells was also not observed. Our data show that JS25 has a potential therapeutic effect in this mouse xenograft model of BL, supported by the generalized reduction in the size of the primary tumors, and in the presence of secondary tumors and metastasis.

### JS25 Demonstrates Selective “On-Target” Activity in the Primary Samples of DLBCL Patients

On the basis of its kinetic and cytotoxic efficiency, we tested the ability of JS25 to induce targeted cell cytotoxicity on viable DLBCL tumor tissues, by collecting lymph node samples from patients with the pathology (Figure 2g). Solid tissues were dissociated; cells were treated with JS25 and ibrutinib, then fixed, and permeabilized; and the resulting monolayers were stained with fluorescent antibodies against surface markers: CD19 (clone HIB19), CD20 (clone L27), and CD79a (clone HM47), along with DAPI. Imaging of the primary cell monolayer was carried out, and the viability and identity (cancer *versus* non-cancer) of individual cells were evaluated using deep learning-driven image analysis. The “on-target” cytotoxicity was identified by calculation of the DRS, which has been shown to correlate with the clinical response for late-stage hematological cancer patients. This score is measured by dividing the fraction of live cancer cells under treatment^35^ by the fraction of live cancer cells of total cells under controls, averaging across multiple concentrations. As shown in Figure 2h and Supporting Figure S2a, JS25 had an “on-target” effect in 7 out of 11 patients (∼64%), and in 4 patients, the “killing” was off-target or nonspecific (∼36%). Ibrutinib presented “on-target” toxicity in 5 out of 10 patients (50%) (Figure 2i and Supporting Figure S2b). Overall, JS25 presented a greater pharmacologic effect at the target of interest than ibrutinib, supported by the number of samples that were more sensitive to the treatment with JS25.

### JS25 is More Effective than Ibrutinib in Zebrafish Patient-Derived Xenografts of CLL

To evaluate the efficacy of JS25 in CLL patient samples, PBMCs were collected and used to generate zebrafish patient-derived xenografts. Here, we compared JS25’s efficacy with ibrutinib’s, and venetoclax was also included as a positive control. Venetoclax is a BH3-mimetic Bcl2 inhibitor that induces significant cell death,^36^ and it is highly efficient for treating CLL; however, the rapid onset of apoptosis often leads to tumor lysis syndrome complications.^37^ In contrast, ibrutinib has different dynamics and therefore is less prone to induce tumor lysis syndrome.^38^

**Figure 3:**
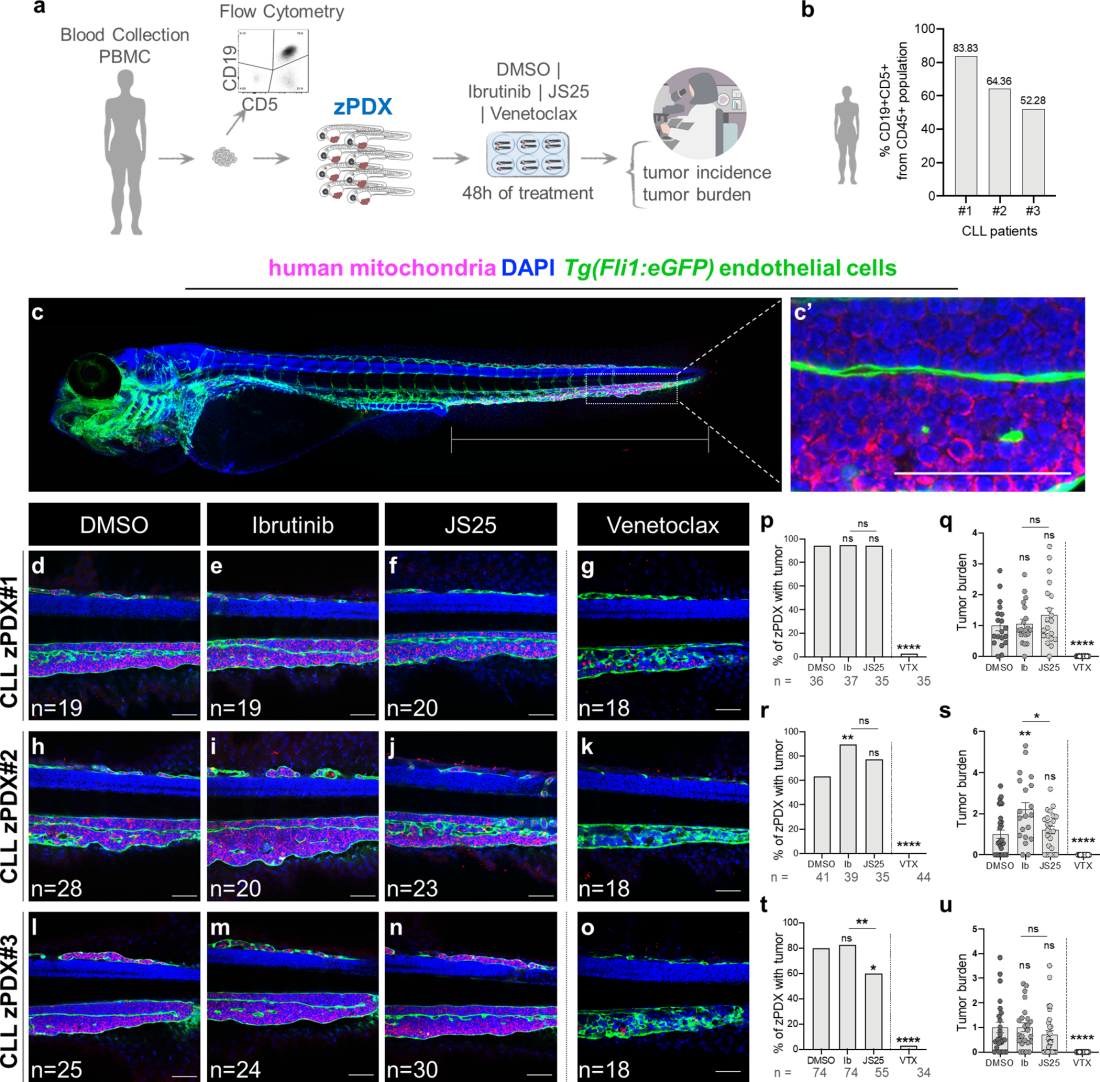
Comparison of the therapeutic effects of BTK inhibitors in zebrafish patient-derived xenografts of CLL disease. (a) Representative scheme of the zPDX assay. (b) Percentage of CD19+CD5+ cells within the CD45+ population from PBMCs of each CLL patient. (c–c′) Representative zPDX confocal image on where the therapeutic effects of the different compounds were analyzed (white rectangle). (d–o) Representative confocal images for each zPDX. Percentage of zPDXs with tumor ((p) ****p < 0.0001, (r) **p = 0.0080, ****p <0.0001, (t) *p = 0.0183, **p = 0.0054, ****p < 0.0001) and tumor burden ((q) ****p <0.0001, (s) *p = 0.0188, **p = 0.0045, ****p <0.0001, (u) ****p < 0.0001). The outcomes are expressed as AVG (b, p, r, t) and AVG ± SEM (fold induction-normalized values to controls) (q, s, u). Data are from one independent experiment, and the number of xenografts analyzed for tumor burden is indicated in the representative images. The number of total zPDXs analyzed at the end of the assay to generate the tumor incidence is indicated below the respective charts. Each dot represents one zebrafish xenograft. Statistical analysis was performed using Fisher’s exact test (tumor incidence) and an unpaired test (tumor burden). Statistical results: ns > 0.05, *p ≤ 0.05, **p ≤ 0.01, ***p ≤ 0.001, and ****p ≤ 0.0001. Scale bar represents 50 μm.

CLL zPDXs with tumor cells in circulation were randomly distributed into four conditions immediately following injection: DMSO (control), ibrutinib (Ib), JS25, and venetoclax. After 48 h of treatment, all zPDXs were fixed and analyzed by confocal microscopy to evaluate tumor burden and incidence (Figure 3a). Tumor incidence is the percentage of zPDXs with tumors by the end of the assay, while tumor burden is the area occupied by PBMCs from the cloaca region until the end of the tail (Figure 3c–c′). In 2 out of the 3 CLL-zPDX, JS25 was more efficient than ibrutinib in reducing the CLL disease burden (Figure 3d–u). In CLL-zPDX#2 (del17p+), JS25 treatment led to a reduction of the tumor burden by ∼45% when compared to ibrutinib (Figure 3h–k,r,s), whereas in CLL-zPDX#3, JS25 reduces the incidence of zPDXs with tumors to 27% relative to ibrutinib and 25% in relation to DMSO controls (Figure 3l–o,t,u) and a tendency to reduce tumor burden (Figure 3l–o,t,u). In all of the zPDXs, venetoclax has a major impact on tumor incidence and burden, being able to induce massive cell death of all CLL cells within 48 h (Figure 3d–u), which is in accordance with the fast CLL cell killing effect observed in patients. Altogether, our results suggest that JS25 has a higher therapeutic impact in CLL, being faster and more effective than its counterpart ibrutinib.

## Discussion

Selective BTK inhibition is well viewed as a promising therapy for multiple hematological cancers and autoimmune diseases. Ibrutinib was the first-in-class BTKi, and although it is well tolerated with a durable response, its clinical use has been limited, prompting the development of second-generation BTKi. Here, we report a new inhibitor, JS25, a covalent small molecule with high potency and selectivity for BTK.

We first characterized the covalent modification of BTK by JS25 using kinetic analysis. An improvement in the covalent binding efficiency of JS25 to BTK was observed when compared to ibrutinib, acalabrutinib, and BMX-IN-1, with an increase of ∼8–200-fold in the rate of protein inactivation (8.72 ± 1.02 μM^–1^ s^–1^). The mechanism of target-specific covalent inhibition is governed by an initial noncovalently binding event that places the reactive electrophile close to the specific nucleophile on the target protein.^30^ The success of this initial fitting dictates the rate of covalent bond formation. Therefore, inhibitors’ structural variations can affect covalent bond formation and consequent target inhibition, as observed in this study. Moreover, the combined effect of higher potency and a faster rate of covalent bond formation seen with JS25 directly translates into less compound required to achieve the same pharmacologic effect, thereby reducing the probability of side effects.^39^

Our MD simulation studies of BTK covalently linked to JS25 demonstrated that Tyr551 was sequestrated around 60% of the whole trajectory, possibly rendering BTK inaccessible for phosphorylation and causing its inactivation. Consistently, inactivation of BTK is usually achieved through blocking of Tyr551 phosphorylation within the Src homology type 1 (SH1) domain by Src kinases, consequently hindering autophosphorylation of Tyr223.^40^ Many BTKi, both covalent and noncovalent, act directly within the SH1 domain, thereby interfering with cell survival and proliferation.

JS25 is also a dual inhibitor of BMX and BTK and presents lower reactivity for TEC, ITK, and TXK and nonreactivity toward EGFR, BLK, JAK3, and Her2. Additionally, we had previously shown that JS25 did not react with other Scr kinases.^23^ On comparing the JS25 selectivity profile with other BTKi (Supporting Table S3), we find that JS25 is less reactive than ibrutinib for TEC, TXK, ITK, EGFR, JAK3, BLK, and Her2; less reactive for TEC and TXK than acalabrutinib; less reactive toward EGFR, JAK3, and Her2 than zanubrutinib; and less reactive toward TEC, TXK, and BLK than tirabrutinib, although slightly more reactive against ITK. The BTKi acalabrutinib, zanubrutinib, and tirabrutinib are second-generation inhibitors, and relative to ibrutinib, these molecules presented fewer “off-target” effects in early clinical trials. Dermatitis is a known adverse side effect attributed to ibrutinib’s “off-targeting” of EGFR;^41^ and bleeding is attributed to the “off-targeting” of the TEC protein, although a recent study suggested it may be caused by inhibition of Scr (*e.g.*, BLK).^42^ In clinical studies, patients treated with BTKi that have no “off-target” effect for TEC kinase (*e.g.*, branebrutinib, evobrutinib, and fenebrutinib) reported less or no bleeding events.^43^ For this reason, it is desirable that newly developed BTKi, such as JS25, have higher selectivity for BTK and lower reactivity toward this particular group of kinases, as shown in this study. The JS25 “off-target” profile suggests a more favorable therapeutic index in comparison to other BTKi. However, some clarification within the clinical context is required to understand whether the JS25 selectivity profile will translate into higher efficacy and safety, particularly in combinatorial regimens with other drugs.

In the cellular context, JS25 presented a wide spectrum of activity against several myeloid/lymphoid B-cell cancers dependent on BTK expression. In addition to inducing degradation of BTK, JS25 effectively crosses the BBB, but with higher retention rates than ibrutinib. However, this does not devaluate the therapeutic potential of JS25 in brain cancers, since higher retention rates can result in longer pharmacological effects, depending on the intracellular metabolism involved. Besides, clinical treatment with acalabrutinib (which showed a similar retention rate to JS25) did not affect the quality of the response to MCL-cell infiltration in the brain.^34^

As a proof of concept of the therapeutic potential, mice with BL were treated with JS25 and presented a reduction of 30–40% in the size of their solid tumors, and an overall reduction in metastasis and secondary tumor formation, relative to ibrutinib. The percentage of metastatic cells present in the liver, lungs, brain/meninges, and spinal cord/bone marrow was similar between treated groups (30% reduction), although lower with a higher JS25 dosage (70% reduction). Naturally, a variety of factors can impact JS25’s distribution throughout the body and even decrease its availability in specific organs.^39^ Thus, within these conditions, a higher drug dosage was more impactful in impairing tumor spread and growth in the mice. Nevertheless, in consistency with the *in vitro* experiments performed here, treatment-induced cell death was significantly more pronounced with JS25. Additionally, no weight fluctuations were observed by the end of the treatment, even at the highest dose (20 mg/kg), suggesting a safe and tolerable profile for JS25 in animal models, within the doses tested.

The drug response score of JS25, in a DLBCL patient model, was slightly higher than that of ibrutinib, proven by the overall increased cell death, leading to 64% “on-target” efficacy. Several genetic variations are on the basis of cellular resistance to ibrutinib in B-cell cancers such as DLBCL,^44^ including the missense cysteine-to-serine mutation at position 481 in BTK, and the compensatory upregulation of the PI3K/AKT signaling pathway. Mutations that lead to acquired resistance to JS25 are still unknown and will be important when evaluating its effectiveness and safety in the clinical stages. Nonetheless, comprehensive drug-responsive profiles such as those generated here directly translate the clinical outcome of JS25 efficacy, thus being a useful route to understand its potential relevance in the clinic.

In the zebrafish patient model of CLL, in 2 out of 3 zPDXs, JS25 was more effective and/or faster than ibrutinib, reducing tumor incidence and tumor burden, thus suggesting a competitive potential of JS25 over ibrutinib as a promising anticancer therapy. CLL is a heterogeneous oncological disease of mature B-cells, in which BTKi are largely prescribed both as first-line and relapse therapy.^45^ The responses to the current FDA- and EMA-approved therapies are diverse and commonly lead to a pathological partial response with incomplete management of the symptoms.^46,47^ Therefore, there is an unmet need to develop more effective and faster BTKi that produce higher antitumoural responses.

## Conclusions

Small-molecule covalent inhibitors combine prolonged inhibition with high selectivity to the target protein. We showed that JS25 binds covalently to BTK at Cys481, and this binding is more efficient than other BTKi in the market. The measurement of selectivity IC_50_ values shows an improved selectivity pattern against EGFR and TEC kinases compared to ibrutinib and the second-generation inhibitors, acalabrutinib, tirabrutinib, and zanubrutinib. JS25 also presented a broad spectrum of activity in myeloid and lymphoid B-cell cancers and demonstrated improved therapeutic efficacy against ibrutinib in patient-derived DLBCL models, as well as in xenograft models of BL and CLL. JS25 also possesses the potential to treat metastatic forms of blood cancers in the brain, as proved by its ability to cross the blood–brain barrier. Taken together, our results establish JS25 as a therapeutically relevant BTKi, with demonstrated antiproliferative effects and improved selectivity profile, and we envisage its clinical use against hematological cancers and autoimmune diseases.

## Supporting Information Available

(1) Maximum tolerated concentration assay performed in zebrafish models; (2) JS25 and ibrutinib’s cytotoxicity in primary DLBCL samples; (3) clinicopathological characterization of CLL patients; (4) stereological analysis of the mice treated with ibrutinib and JS25; (5) heat map with selectivity screening values of several BTK inhibitors (PDF)

## Notes

J.D.S. and G.J.L.B. are inventors in a patent (WO2020245430A1) related to the findings reported in this manuscript. Other authors declare no competing interests.

## Notes

All animal studies were conducted under the approval of Direção Geral da Alimentação e Veterinária (DGAV), following the European Animal Welfare Legislation (Directive 2010/63/EU). Human tissue collection for DLBCL patient models was conducted in accordance with the Declaration of Helsinki, and the protocol was approved by the Ethics Committee of the Medical University of Graz (Ethical application 30-528 ex 17/18). For human tissue collection in zebrafish xenograft model studies, informed consent was obtained from all of the participants, and the experiments were conducted according to the Ethics Committee at Champalimaud Foundation and following hospital standard operating procedures.

## Abbreviations

BTK: Bruton’s tyrosine kinase
AML: acute myeloid leukemia
ALL: acute lymphocytic leukemia
CLL: chronic lymphocytic leukemia
WM: Waldenström’s macroglobulinaemia
MCL: mantle cell lymphoma
BL: Burkitt’s lymphoma
DLBCL: diffuse large B-cell lymphoma
BTKi: BTK inhibitors
MD: Molecular dynamics
HBEC-5i: human cerebral microvascular endothelial cells
DRS: drug response score
PBMCs: peripheral blood mononuclear cells isolation
zPDX: zebrafish patient-derived xenograft
*K*_I_: initial noncovalent binding
*k*_inact_: rate of covalent bond formation
*k*_inact_/*K*_I_: rate of inactivation
APML: acute promyelocytic leukemia
CNSi: central nervous system
Ib: ibrutinib
