# Supplementary material for: Selective Inhibition of Bruton’s Tyrosine Kinase by a Designed Covalent Ligand Leads to Potent Therapeutic Efficacy in Blood Cancers Relative to Clinically Used Inhibitors
Source: ACS Pharmacol Transl Sci. 2022 Nov 2;5(11):1156–68. doi: 10.1021/acsptsci.2c00163 (PMC9667546; doi:10.1021/acsptsci.2c00163)
Supplement: Supplementary file 1 — pt2c00163_si_001.pdf [file pt2c00163_si_001.pdf]

Selective inhibition of Bruton's tyrosine kinase by a designed covalent ligand leads to potent therapeutic efficacy in blood cancers relative to clinically used inhibitors

*Bárbara B. Sousa[1], Cátia Rebelo de Almeida[2], Ana F. Barahona[2], Raquel Lopes[2], Ana Martins-Logrado[2], Marco Cavaco[1], Vera Neves[1], Luís A. R. Carvalho[3], Carlos Labão-Almeida[1], Ana R. Coelho[1], Marta Leal Bento[1,4], Ricardo M. R. M. Lopes[5], Bruno L. Oliveira[1], Miguel A. R. B. Castanho[1], Peter Neumeister[6], Alexander Deutsch[6], Gregory I. Vladimer[7], Nikolaus Krall[7], Cristina João[2], Francisco Corzana[8], João D. Seixas[1,9], Rita Fior[2], Gonçalo J. L. Bernardes[1,3]**

[1]Instituto de Medicina Molecular João Lobo Antunes, Faculdade de Medicina, Universidade de Lisboa, Avenida Prof. Egas Moniz, 1649-028, Lisbon, Portugal.

[2]Champalimaud Foundation, Avenida de Brasília, 1400-038, Lisbon, Portugal.

[3]Yusuf Hamied Department of Chemistry, University of Cambridge, Lensfield Road, Cambridge, CB2 1EW, UK.

[4]Centro Hospitalar Lisboa Norte, Department of Hematology and Bone Marrow Transplantation, Avenida Prof. Egas Moniz, 1649-035, Lisbon, Portugal.

[5]Research Institute for Medicines (iMed.ULisboa), Faculdade de Farmácia, Universidade de Lisboa, 1600-277, Lisbon, Portugal.

[6]Division of Hematology, Medical University of Graz, Auenbruggerplatz 38, 8036 Graz, Austria.

[7]Exscientia, The Schrödinger Building, Oxford Science Park, Oxford, OX4 4GE, UK.

[8]Centro de Investigación en Síntesis Química, Departamento de Química, Universidad de La Rioja, 26006 Logroño, Spain.

[9]TARGTEX S.A., Avenida Tenente Valadim, Nº17, 2F, 2560-275, Torres Vedras, Portugal.

***Corresponding author: Gonçalo J. L. Bernardes, gb453@cam.ac.uk**

Table of contents

SUPPORTING FIGURES AND TABLES……………........……………………………………………………S1
Figure S1. Maximum tolerated concentration assay. S1
Figure S2. *Ex vivo* cytotoxicity in primary DLBCL samples.………………....………….......……………………..S1
Table S1. Clinico-pathological characterisation of CLL patients. S2
Table S2. Stereological analysis of the mice treated with Ibrutinib and JS25. .... S2
Table S3. Heat map with selectivity screening values. S3
REFERENCES S4

SUPPORTING FIGURES AND TABLES

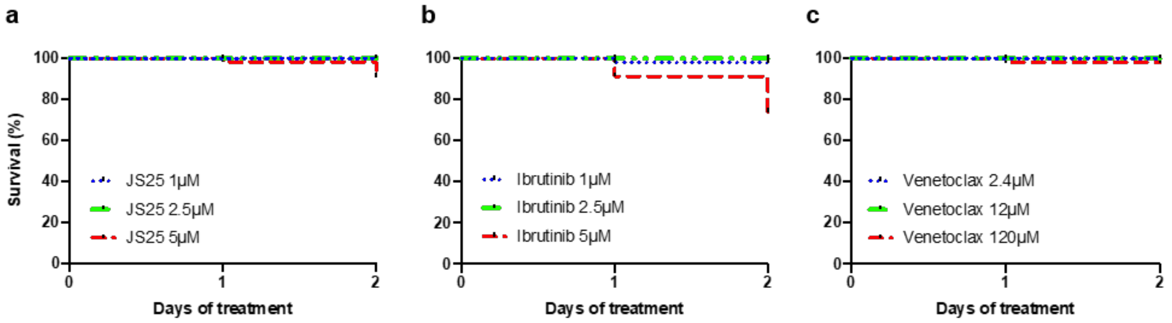

Figure S1. Maximum tolerated concentration assay. Starting at 2 days post-fertilisation, different groups of random non-injected zebrafish larvae were exposed to different concentrations of JS25 (**a**), Ibrutinib (**b**) and Venetoclax (**c**). Graphs represent the zebrafish survival at different concentrations of JS25, Ibrutinib and Venetoclax.

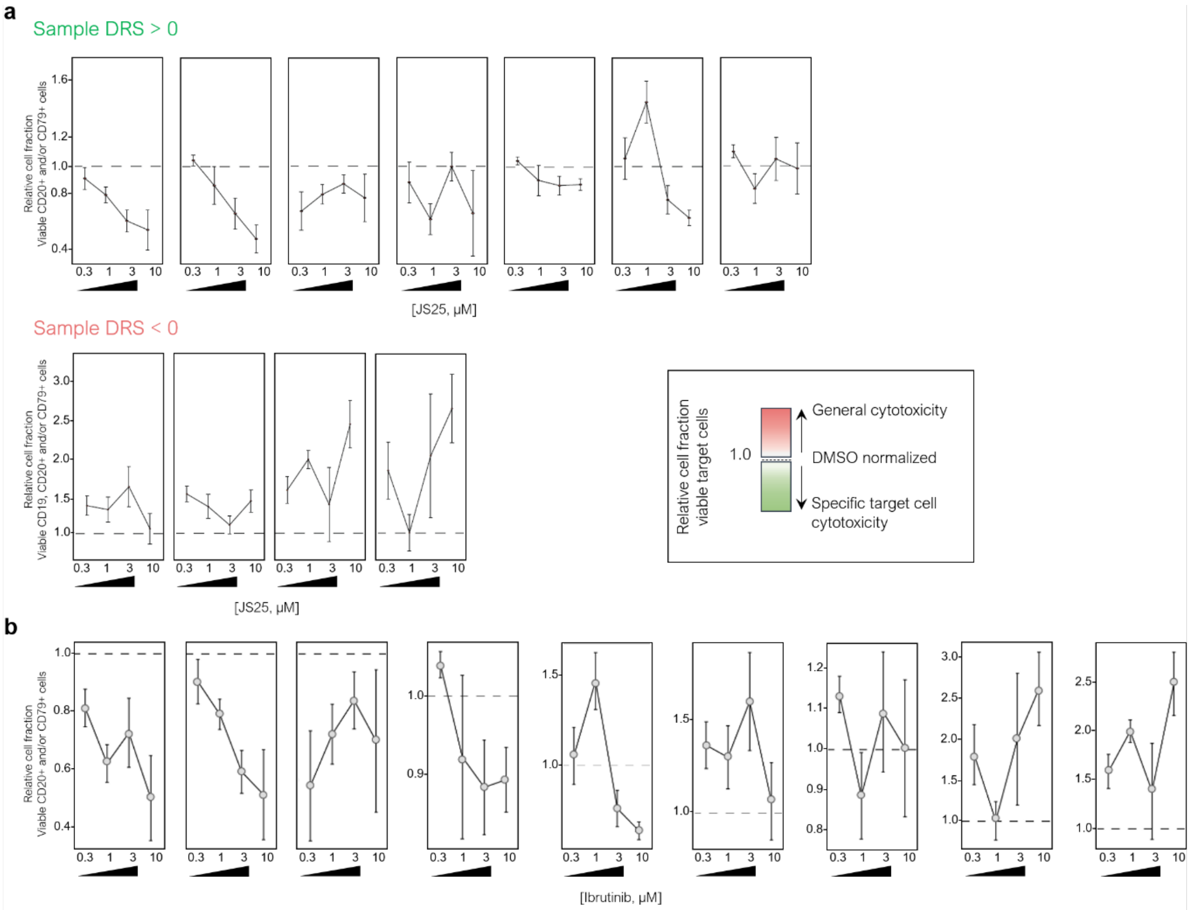

Figure S2. *Ex vivo* cytotoxicity in primary DLBCL samples. (a) Fraction of viable target cancer cells for increasing concentrations of JS25 relative to DMSO ("Relative Cell Fraction" or RCF); RCF < 1 indicates a selective anticancer effect over non-cancerous cells. RCF > 1 stronger toxicity against non-cancer than cancer cells. Green indicates a DRS > 0, red indicates DRS < 0. DRS scores > 0 indicates "on-target" cytotoxic response, < 0 indicates general cytotoxicity or "off-target" cytotoxic response. Positive DRS have previously been shown to associate with prolonged progression free survival. Data are shown as mean ± SE shown, each concentration point for each sample was performed in 4 replicates, at a single 72 h hour incubation time point. (b) Relative cell fraction (RCF) of the viable target cells for Ibrutinib and DMSO.

Table S1. Clinico-pathological characterisation of CLL patients.

CLL Patient	Gender/ Age	CLL-IPI Score (Risk Group)	Binet/Rai Staging	β2-microglobulin [mg/L]	IGHV mutations	Patient status at sampling	Citogenetic alterations
#1	M/75	n.a.*	A/0	Unknown	Unknown	Naïve."Watch and wait" since 2013.	Unknown
#2	M/69	7 (Very High)	A/0	7,24	No	Venetoclax interruption due to severe infection. Partial Response (5th line).	del17p+
#3	M/73	n.a.*	B/II	Unknown	Unknown	Naïve. "Watch and wait"since February 2021.	Unknown

M - male; n.a.* not applicable to patients not eligible for treatment

Table S2. Stereological analysis of the mice treated with Ibrutinib and JS25.[a]

ANIMAL[b]	Metastasis volume (mm^3)				Secondary tumour (mm^3)
	Liver	Brain	Spinal	Lung	
Control #1	0.61	0.93	39.60	0.00	76.82
Control #2	10.46	1.96	51.36	0.00	290.14
Control #3	0.00	0.14	42.00	0.00	84.63
Control #4	10.58	1.21	71.85	0.00	82.41
Control #5	6.08	0.23	59.40	88.59	195.39
**Average**	**5.55**	**0.89**	**52.84**	**17.72**	**145.88**
**St.D**	**4.58**	**0.67**	**11.83**	**35.43**	**84.63**
Ibrutinib #1	0.00	0.48	99.35	0.00	96.59
Ibrutinib #2	12.05	0.86	55.71	134.59	0.00
Ibrutinib #3	11.54	0.82	86.64	0.00	224.46
Ibrutinib #4	0.03	0.00	4.378	0.00	0.00
Ibrutinib #5	0.00	1.72	10.76	0.00	0.00
**Average**	**4.73**	**0.78**	**51.37**	**26.92**	**64.21**
**St.D**	**5.78**	**0.56**	**38.53**	**53.84**	**88.43**
JS25(10) #1	0.00	0.92	18.31	0.00	0.00
JS25(10) #2	9.94	0.98	109.42	0.00	0.00
JS25(10) #3	83.71	2.33	122.01	130.18	84.38
JS25(10) #4	43.05	0.15	88.70	0.00	0.00
JS25(10) #5	0.00	1.27	98.09	0.00	0.00
**Average**	**27.34**	**1.13**	**87.31**	**26.04**	**16.88**
**St.D**	**32.32**	**0.71**	**36.25**	**52.07**	**33.75**
JS25(20) #1	0.00	0.44	36.97	0.00	0.00
JS25(20) #2	2.00	0.03	12.59	0.00	91.12
JS25(20) #3	3.35	0.82	70.62	0.00	86.92
JS25(20) #4	0.64	0.00	14.65	0.00	8.82
JS25(20) #5	0.70	0.00	0.69	0.00	3.06
**Average**	**1.34**	**0.26**	**27.10**	**0.00**	**37.98**
**St.D**	**1.20**	**0.33**	**24.72**	**0.00**	**41.79**

[a]Neoplastic cells in the heart, kidney, bone marrow of the ribs, femur and pelvis, were excluded from the calculation, as these were not observed in totality and consistently in all animals. [b]JS25(10) – JS25 10 mg/Kg; JS25(20) – JS25 20 mg/Kg.

Table S3. Heat map with selectivity screening values.

	BTK	BMX	TEC	TXK	ITK	EGFR	BLK	JAK3	Her2	Status
Ibrutinib	1.5	0.8	7	2	62	2	0.1	32	36	CLL, MCL, WM, NHL, cGVHD – FDA approv[1,2]
Acalabrutinib	5.1	46	93	368	>1000	>1000	>1000	>1000	1000	CLL, MCL – FDA approv; WM – Phase 2[3,4]
Zanubrutinib	0.22	Not tested	1.9	Not tested	30	660	Not tested	200	661	MCL – FDA approv; CLL, WM – Phase 3[3,5]
Tirabrutinib	6.8	6	48	92	> 20000	3020	300	5515	7313	WM – approv in Japan; CLL, RA, NHL – Phase 1/2[6,7]
Evobrutinib	8.9 (90%)	93%	82%	36%	13%	0%	36%	0%	Not tested	MS – Phase 3[8–10]
TG-1701	3	~1000	4	136	>3000	270	~1000	>3000	>3000	MCL, WM, CLL, DLBCL – Phase 1/2[11,12]
Branebrutinib	0.1	1.5	0.9	5	100	Not tested	Not tested	>500	Not tested	RA, Lupus – Phase 2[13]
Vecabrutinib	3	224	14	474	14	>6000	23	Not tested	Not tested	FL, WM, CLL, DLBCL, MCL – Phase 2[14–16]
Fenebrutinib	2.3	351	>1000	>1000	>1000	>1000	>1000	>1000	>1000	RA, Lupus – Phase 2; FL, CLL, MCL, DLBCL – Phase 1; MS – Phase 3[17–19]
**JS25**[20]	5.8/28	3.5/49	220	190	440	> 3000	~3000	> 3000	> 3000	

**IC_{50}/nM**	**< 10**	**11-100**	**101-500**	**501-1000**	**> 1000**

*1 µM inhibition screening; **non-covalent; approv – approved; CLL – chronic lymphocytic leukaemia; MCL – mantle cell lymphoma; WM – Waldenstrom macroglobulinemia; NHL – Non-Hodgkin lymphoma; cGVHD – chronic graft-versus-host disease; RA – Rheumatoid arthritis; MS – multiple sclerosis; FL – Follicular lymphoma; DLBCL – diffuse-large B-cell lymphoma.

REFERENCES

(1) Honigberg, L. A.; Smith, A. M.; Sirisawad, M.; Verner, E.; Loury, D.; Chang, B.; Li, S.; Pan, Z.; Thamm, D. H.; Miller, R. A.; Buggy, J. J. The Bruton Tyrosine Kinase Inhibitor PCI-32765 Blocks B-Cell Activation and Is Efficacious in Models of Autoimmune Disease and B-Cell Malignancy. *Proc. Natl. Acad. Sci. U.S.A.* **2010**, *107* (29), 13075–13080. DOI: 10.1073/pnas.1004594107.

(2) Pan, Z.; Scheerens, H.; Li, S. J.; Schultz, B. E.; Sprengeler, P. A.; Burrill, L. C.; Mendonca, R. v.; Sweeney, M. D.; Scott, K. C. K.; Grothaus, P. G.; Jeffery, D. A.; Spoerke, J. M.; Honigberg, L. A.; Young, P. R.; Dalrymple, S. A.; Palmer, J. T. Discovery of Selective Irreversible Inhibitors for Bruton's Tyrosine Kinase. *ChemMedChem* **2007**. DOI: 10.1002/cmdc.200600221.

(3) Estupiñán, H. Y.; Berglöf, A.; Zain, R.; Smith, C. I. E. Comparative Analysis of BTK Inhibitors and Mechanisms Underlying Adverse Effects. *Front. Cell Dev. Biol.* **2021**, *9*. DOI: 10.3389/fcell.2021.630942.

(4) Barf, T.; Covey, T.; Izumi, R.; van de Kar, B.; Gulrajani, M.; van Lith, B.; van Hoek, M.; de Zwart, E.; Mittag, D.; Demont, D.; Verkaik, S.; Krantz, F.; Pearson, P. G.; Ulrich, R.; Kaptein, A. Acalabrutinib (ACP-196): A Covalent Bruton Tyrosine Kinase Inhibitor with a Differentiated Selectivity and In Vivo Potency Profile. *J. Pharmacol. Exp. Ther.* **2017**, *363* (2), 240–252. DOI: 10.1124/jpet.117.242909.

(5) Flinsenberg, T. W. H.; Tromedjo, C. C.; Hu, N.; Liu, Y.; Guo, Y.; Thia, K. Y. T.; Noori, T.; Song, X.; Aw Yeang, H. X.; Tantalo, D. G.; Handunnetti, S.; Seymour, J. F.; Roberts, A. W.; Ritchie, D.; Koldej, R.; Neeson, P. J.; Wang, L.; Trapani, J. A.; Tam, C. S.; Voskoboinik, I. Differential Effects of BTK Inhibitors Ibrutinib and Zanubrutinib on NK-Cell Effector Function in Patients with Mantle Cell Lymphoma. *Haematologica* **2020**, *105* (2), e76–e79. DOI: 10.3324/haematol.2019.220590.

(6) Liclican, A.; Serafini, L.; Xing, W.; Czerwieniec, G.; Steiner, B.; Wang, T.; Brendza, K. M.; Lutz, J. D.; Keegan, K. S.; Ray, A. S.; Schultz, B. E.; Sakowicz, R.; Feng, J. Y. Biochemical Characterization of Tirabrutinib and Other Irreversible Inhibitors of Bruton's Tyrosine Kinase Reveals Differences in on - and off - Target Inhibition. *Biochim. Biophys. Acta Gen. Subj.* **2020**, *1864* (4), 129531. DOI: 10.1016/j.bbagen.2020.129531.

(7) Dhillon, S. Tirabrutinib: First Approval. *Drugs* **2020**, *80* (8), 835–840. DOI: 10.1007/s40265-020-01318-8.

(8) Haselmayer, P.; Camps, M.; Liu-Bujalski, L.; Nguyen, N.; Morandi, F.; Head, J.; O'Mahony, A.; Zimmerli, S. C.; Bruns, L.; Bender, A. T.; Schroeder, P.; Grenningloh, R. Efficacy and Pharmacodynamic Modeling of the BTK Inhibitor Evobrutinib in Autoimmune Disease Models. *J. Immunol.* **2019**, *202* (10), 2888–2906. DOI: 10.4049/jimmunol.1800583.

(9) Caldwell, R. D.; Qiu, H.; Askew, B. C.; Bender, A. T.; Brugger, N.; Camps, M.; Dhanabal, M.; Dutt, V.; Eichhorn, T.; Gardberg, A. S.; Goutopoulos, A.; Grenningloh,

R.; Head, J.; Healey, B.; Hodous, B. L.; Huck, B. R.; Johnson, T. L.; Jones, C.; Jones, R. C.; Mochalkin, I.; Morandi, F.; Nguyen, N.; Meyring, M.; Potnick, J. R.; Santos, D. C.; Schmidt, R.; Sherer, B.; Shutes, A.; Urbahns, K.; Follis, A. V.; Wegener, A. A.; Zimmerli, S. C.; Liu-Bujalski, L. Discovery of Evobrutinib: An Oral, Potent, and Highly Selective, Covalent Bruton's Tyrosine Kinase (BTK) Inhibitor for the Treatment of Immunological Diseases. *J. Med. Chem.* **2019**, *62* (17), 7643–7655. DOI: 10.1021/acs.jmedchem.9b00794.

(10) Scheible, H.; Dyroff, M.; Seithel-Keuth, A.; Harrison-Moench, E.; Mammasse, N.; Port, A.; Bachmann, A.; Dong, J.; Lier, J. J.; Tracewell, W.; Mitchell, D. Evobrutinib, a Covalent Bruton's Tyrosine Kinase Inhibitor: Mass Balance, Elimination Route, and Metabolism in Healthy Participants. *Clin. Transl. Sci.* **2021**, *14* (6), 2420–2430. DOI: 10.1111/cts.13108.

(11) Cheah, C. Y.; Jurczak, W.; Lasica, M.; Wróbel, T.; Cheung, S.; Walewski, J.; Giannopoulos, K.; Yannakou, C. K.; Lewis, K. L.; Dlugosz-Danecka, M.; Miskin, H. P.; Ricart, A. D.; O'Connor, O. A.; Tam, C. S. The Selective Bruton Tyrosine Kinase (BTK) Inhibitor TG-1701 As Monotherapy and in Combination with Ublituximab and Umbralisib (U2) in Patients with B-Cell Malignancies. *Blood* **2021**, *138* (Supplement 1), 1549–1549. DOI: 10.1182/blood-2021-145911.

(12) Ribeiro, M. L.; Reyes-Garau, D.; Vinyoles, M.; Profitós Pelejà, N.; Santos, J. C.; Armengol, M.; Fernández-Serrano, M.; Sedó Mor, A.; Bech-Serra, J. J.; Blecua, P.; Musulen, E.; de La Torre, C.; Miskin, H.; Esteller, M.; Bosch, F.; Menéndez, P.; Normant, E.; Roué, G. Antitumor Activity of the Novel BTK Inhibitor TG-1701 Is Associated with Disruption of Ikaros Signaling in Patients with B-Cell Non–Hodgkin Lymphoma. *Clin. Cancer Res.* **2021**, *27* (23), 6591–6601. DOI: 10.1158/1078-0432.CCR-21-1067.

(13) Watterson, S. H.; Liu, Q.; Beaudoin Bertrand, M.; Batt, D. G.; Li, L.; Pattoli, M. A.; Skala, S.; Cheng, L.; Obermeier, M. T.; Moore, R.; Yang, Z.; Vickery, R.; Elzinga, P. A.; Discenza, L.; D'Arienzo, C.; Gillooly, K. M.; Taylor, T. L.; Pulicicchio, C.; Zhang, Y.; Heimrich, E.; McIntyre, K. W.; Ruan, Q.; Westhouse, R. A.; Catlett, I. M.; Zheng, N.; Chaudhry, C.; Dai, J.; Galella, M. A.; Tebben, A. J.; Pokross, M.; Li, J.; Zhao, R.; Smith, D.; Rampulla, R.; Allentoff, A.; Wallace, M. A.; Mathur, A.; Salter-Cid, L.; Macor, J. E.; Carter, P. H.; Fura, A.; Burke, J. R.; Tino, J. A. Discovery of Branebrutinib (BMS-986195): A Strategy for Identifying a Highly Potent and Selective Covalent Inhibitor Providing Rapid in Vivo Inactivation of Bruton's Tyrosine Kinase (BTK). *J. Med. Chem.* **2019**, *62* (7), 3228–3250. DOI: 10.1021/acs.jmedchem.9b00167.

(14) Gu, D.; Tang, H.; Wu, J.; Li, J.; Miao, Y. Targeting Bruton Tyrosine Kinase Using Non-Covalent Inhibitors in B Cell Malignancies. *J. Hematol. Oncol.* **2021**, *14* (1), 40. DOI: 10.1186/s13045-021-01049-7.

(15) Jebaraj, B. M. C.; Müller, A.; Dheenadayalan, R. P.; Endres, S.; Roessner, P. M.; Seyfried, F.; Walliser, C.; Wist, M.; Qi, J.; Tausch, E.; Mertens, D.; Fox, J. A.; Debatin, K.-M.; Meyer, L. H.; Taverna, P.; Seiffert, M.; Gierschik, P.; Stilgenbauer, S. Evaluation of Vecabrutinib as a Model for Noncovalent BTK/ITK Inhibition for Treatment of

Chronic Lymphocytic Leukemia. *Blood* **2022**, *139* (6), 859–875. DOI: 10.1182/blood.2021011516.

(16) Allan, J. N.; Pinilla-Ibarz, J.; Gladstone, D. E.; Patel, K.; Sharman, J. P.; Wierda, W. G.; Choi, M. Y.; O'Brien, S. M.; Shadman, M.; Davids, M. S.; Pagel, J. M.; Yimer, H. A.; Ward, R.; Acton, G.; Taverna, P.; Combs, D. L.; Fox, J. A.; Furman, R. R.; Brown, J. R. Phase 1b Dose-Escalation Study of the Selective, Noncovalent, Reversible Bruton's Tyrosine Kinase Inhibitor Vecabrutinib in B-Cell Malignancies. *Haematologica* **2021**. DOI: 10.3324/haematol.2021.280061.

(17) Byrd, J. C.; Smith, S.; Wagner-Johnston, N.; Sharman, J.; Chen, A. I.; Advani, R.; Augustson, B.; Marlton, P.; Renee Commerford, S.; Okrah, K.; Liu, L.; Murray, E.; Penuel, E.; Ward, A. F.; Flinn, I. W. First-in-Human Phase 1 Study of the BTK Inhibitor GDC-0853 in Relapsed or Refractory B-Cell NHL and CLL. *Oncotarget* **2018**, *9* (16), 13023–13035. DOI: 10.18632/oncotarget.24310.

(18) Xu, H.; Jesson, M. I.; Seneviratne, U. I.; Lin, T. H.; Sharif, M. N.; Xue, L.; Nguyen, C.; Everley, R. A.; Trujillo, J. I.; Johnson, D. S.; Point, G. R.; Thorarensen, A.; Kilty, I.; Telliez, J.-B. PF-06651600, a Dual JAK3/TEC Family Kinase Inhibitor. *ACS Chem. Biol.* **2019**, *14* (6), 1235–1242. DOI: 10.1021/acschembio.9b00188.

(19) Ringheim, G. E.; Wampole, M.; Oberoi, K. Bruton's Tyrosine Kinase (BTK) Inhibitors and Autoimmune Diseases: Making Sense of BTK Inhibitor Specificity Profiles and Recent Clinical Trial Successes and Failures. *Front. Immunol.* **2021**, *12*, 662223. DOI: 10.3389/fimmu.2021.662223.

(20) Seixas, J. D.; Sousa, B. B.; Marques, M. C.; Guerreiro, A.; Traquete, R.; Rodrigues, T.; Albuquerque, I. S.; Sousa, M. F. Q.; Lemos, A. R.; Sousa, P. M. F.; Bandeiras, T. M.; Wu, D.; Doyle, S. K.; Robinson, C. v.; Koehler, A. N.; Corzana, F.; Matias, P. M.; Bernardes, G. J. L. Structural and Biophysical Insights into the Mode of Covalent Binding of Rationally Designed Potent BMX Inhibitors. *RSC Chem. Biol.* **2020**, *1* (4), 251–262. DOI: 10.1039/D0CB00033G.
